# First case report of polymyxin B-induced drug fever in a patient with carbapenem-resistant *Acinetobacter baumannii* pneumonia

**DOI:** 10.3389/fphar.2026.1812802

**Published:** 2026-06-16

**Authors:** Ying Liu, Xiang Liu, Li Zou, Chen Wang, Xing-Xing Xu, Li Min, Pei Guo

**Affiliations:** 1 Department of Basic Medical Science, Chongqing Three Gorges Medical College, Chongqing, China; 2 Department of Pharmacy, The First Affiliated Hospital of Chongqing Medical and Pharmaceutical College, Chongqing, China; 3 Intensive Care Unit, The First Affiliated Hospital of Chongqing Medical and Pharmaceutical College, Chongqing, China

**Keywords:** adverse drug reaction, carbapenem-resistant *Acinetobacter* baumannii, drug fever, polymyxin B, severe pneumonia

## Abstract

The escalating global prevalence of carbapenem-resistant Gram-negative bacteria has significantly constrained therapeutic options, leading to the revived use of polymyxins. However, this resurgence has been paralleled by an increase in reports of associated adverse effects. No case of drug fever induced by polymyxin B (PMB) has been reported in the literature to date. We report the first case of drug fever (≥39 °C) induced by intravenous PMB in a patient with severe pneumonia caused by carbapenem-resistant *Acinetobacter baumannii*, accompanied by tachypnea following concomitant nebulized PMB administration. Upon intervention by the clinical pharmacist, which involved discontinuation of PMB and adjustment of the anti-infective regimen, the patient’s fever resolved promptly and the infection was successfully controlled. This case highlights a severe and previously unreported adverse drug reaction to PMB, underscoring the necessity for vigilant monitoring and proactive antimicrobial stewardship in managing infections.

## 1 Introduction

Polymyxin antibiotics were first introduced into clinical use in the 1950s but were subsequently phased out due to significant nephrotoxicity and neurotoxicity. The escalating global crisis of carbapenem-resistant Gram-negative bacteria (CR-GNB) has necessitated their revival as a critical last-line therapeutic option (Cepas and Soto, 2020). These agents are primarily deployed against CR-GNB, including *K. pneumoniae* (*Klebsiella pneumoniae*), *Escherichia coli* (*E. coli*), *Pseudomonas aeruginosa*, and *Acinetobacter baumannii*. According to the latest China hospital infection monitoring network (CHINET) surveillance data from the first half of 2025 (CHINET, 2025), the resistance rate to polymyxin B (PMB) against these CR-GNB remains relatively low, ranging from 0.7% to 11%, and the resistance rate to carbapenem-resistant *A. baumannii* (CRAB) was 0.8%. Current international guidelines strongly recommend that PMB be used in combination with other agents, such as sulbactam, carbapenems, or novel β-lactam/β-lactamase inhibitor combinations, to mitigate the risk of heteroresistance and enhance bacterial eradication (Zhang et al., 2017; Zusman et al., 2017). For pulmonary CR-GNB infections, a dual-route strategy combining intravenous administration with nebulization is advocated to achieve higher alveolar drug concentrations, accelerate bacterial clearance, and potentially shorten the duration of mechanical ventilation (American Thoracic Society, 2005). However, these intensified regimens—both combination therapy and dual-route delivery—inherently increase the risk of adverse drug reactions (ADRs).

The toxicity profile of PMB is predominantly characterized by a triad of nephro-, dermato-, and neurotoxicity. Beyond these common adverse effects, rarer ADRs have been documented, including respiratory paralysis (Ning et al., 2022), respiratory failure (Chin et al., 2024), anaphylactic shock (Liu et al., 2025), Bartter-like syndrome (Taik et al., 2024), and rhabdomyolysis (Ni et al., 2020). Notably, while drug fever is listed as a potential reaction in the package insert, no confirmed case report of PMB-induced drug fever has been published to date. Drug fever is a distinct hypersensitivity reaction characterized by fever that emerges during drug exposure, resolves upon its discontinuation, and for which other causes have been excluded. Its onset typically occurs 3–20 days after initiation of therapy, though it can manifest as early as 2 h post-dose. The fever pattern (≥38.5 °C) may be remittent, continuous, or intermittent, and typically subsides within 2–48 h after drug withdrawal (Someko et al., 2024). Laboratory findings, such as mild elevations in white blood cell count (WBC) or C-reactive protein (CRP), are non-specific. However, eosinophilia is more representative. Among its various causes, antimicrobial-induced drug fever is particularly underrecognized, as it is notoriously difficult to distinguish from infectious fever. This diagnostic challenge can lead to inappropriate antimicrobial escalation, increased risk of resistance, prolonged patient morbidity, and unnecessary healthcare costs.

To our knowledge (Chen and Wang, 2025), this report describes the first confirmed case of high-grade drug fever induced by PMB therapy to date.

## 2 Case description

A 63-year-old male patient was transferred to our tertiary care general hospital 27 days following a motor vehicle accident. His initial presentation at the referring hospital was managed for severe polytrauma, including traumatic brain injury (multiple intracranial hematomas, diffuse axonal injury, traumatic subarachnoid hemorrhage), multiple fractures (bilateral mandible, bilateral humeri, multiple ribs, T11 and L1-L3 vertebrae, right pubis, calcaneus, and talus), bilateral pulmonary contusions, and hemopneumothorax. During this initial hospitalization, he developed a hospital-acquired pneumonia. Bronchoalveolar lavage culture grew *S. aureus*, and metagenomic next-generation sequencing analysis confirmed *Staphylococcus aureus* and additionally identified *Enterobacter cloacae*, *K. pneumoniae*, and *E. coli*. His antimicrobial therapy had been escalated sequentially with oxacillin, levofloxacin, minocycline, vancomycin, and ceftazidime, without achieving full clinical resolution. Upon admission to our unit, the patient remained comatose with persistent pneumonia. On day 2, he spiked a fever of 38.8 °C, prompting initiation of empirical piperacillin-tazobactam. Thoracic drainage fluid on day 4 grew an extended-spectrum beta-lactamase-producing *E. coli*, and sputum culture on day 11 yielded CRAB. Following a recommendation from the clinical pharmacist, the regimen was switched to tigecycline combined with meropenem.

**FIGURE 1:**
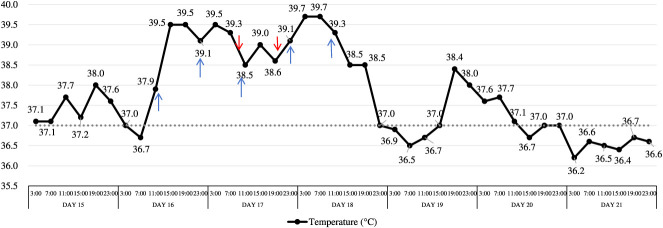
Detailed temperature trend over 1 week following PMB-induced drug fever. Note: Blue arrows, intravenous PMB infusion; red arrows, nebulized PMB administration.

On day 16, the infection remained uncontrolled. The patient had a persistent fever (38 °C on day 15), copious sputum production, oxygenation deficit, elevated inflammatory markers, and a repeat BAL confirmed co-infection with multidrug-resistant *K. pneumoniae* and CRAB. Given the limited therapeutic options and the institution’s antimicrobial formulary, the clinical pharmacist recommended a last-line salvage regimen: intravenous PMB at 500,000 IU every 12 h combined with cefoperazone-sulbactam (1:1) at 3 g every 8 h, with the addition of nebulized PMB (250,000 IU every 12 h) if deemed necessary. The first intravenous PMB dose was administered at 11:00 on day 16. Notably, the patient’s temperature, which was 37.9 °C prior to PMB infusion, began to rise steadily following the initiation of the infusion and peaked at 39.5 °C by 15:00 the same day (Figure 1). This high-grade fever was refractory to physical cooling and ibuprofen. While his blood pressure and respiratory rate remained relatively stable, raising initial suspicion for central fever, subsequent electroencephalogram showed severe abnormalities. On day 17, nebulized PMB was added to the regimen. However, the high-grade fever persisted without improvement in infection biomarkers. Suspecting uncontrolled infection, cefoperazone-sulbactam was switched to ceftazidime-avibactam (2.5 g every 8 h) that evening. On day 18, with the patient’s fever unabated for over 48 h, the clinical pharmacist conducted a formal temporal analysis. A critical dissociation was noted: the persistent, high-grade fever demonstrated a poor correlation with trends in procalcitonin, white blood cell count, and respiratory symptoms. This discrepancy led to the hypothesis of a drug fever, prompting the recommendation to discontinue PMB. The final intravenous dose of PMB was administered at 11:00 that day, but the scheduled nebulization was withheld. It was later noted by the clinical team that the patient’s respiratory rate had increased during previous nebulized PMB sessions, peaking above 30 breaths per minute, though this fluctuation had initially been attributed to his underlying condition rather than a potential ADR. The clinical team considered differential diagnoses for the fever, including methicillin-resistant *S. aureus* infection. Vancomycin was initiated, with the clinical pharmacist providing model-informed precision dosing recommendations. However, after five doses, the patient developed acute kidney injury, potentially attributable to vancomycin, prompting its discontinuation. Crucially, the patient’s temperature dropped to 37.0 °C by 23:00 on day 18, following the last PMB dose. The fever markedly subsided thereafter, with only occasional low-grade temperatures observed (Figure 1).

**FIGURE 2:**
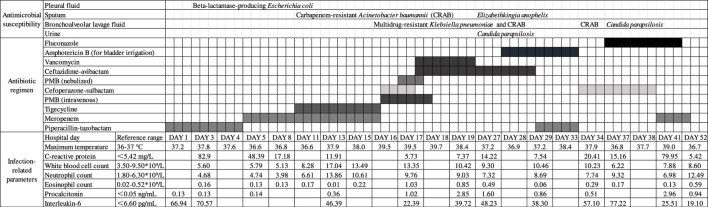
Timeline of antimicrobial therapy and key infection parameters.

The pulmonary infection was controlled following a 12-day course of ceftazidime-avibactam, without recurrence of high-grade fever. Subsequent complications included a urinary tract infection with *Candida parapsilosis*, managed with amphotericin B bladder irrigation, and pulmonary infections with *Elizabethkingia anophelis* and *C. parapsilosis*, treated with piperacillin-tazobactam, cefoperazone sulbactam and fluconazole. A final 12-day course of meropenem was administered. All antimicrobials were discontinued on day 52, marking control of the infectious complications, and the patient transitioned to rehabilitation. The overall anti-infection process and related indicators of the patient are shown in Figure 2.

## 3 Discussion

This case report presents the first documented instance of drug fever induced by PMB, with the Suspected drug discontinued at the earliest possible time. It highlights the potential for PMB to cause drug fever in clinical practice, underscoring the necessity of differentiating it from infectious fever and maintaining a high index of clinical suspicion. According to the Naranjo Adverse Drug Reaction Probability Scale, the association between PMB and the drug fever in this case was rated as “probable,” with a score of 8. The temporal relationship was clear: the patient’s fever manifested following PMB infusion, with a gradual temperature rise initiating post-infusion, peaking at 4 h, and subsiding after drug discontinuation, returning to normal within 12 h. No temporal correlation or rechallenge positivity was observed with other concomitant medications. No high fever was observed during subsequent use of cefoperazone-sulbactam or meropenem. While CRP, WBC, and neutrophil count were not indicative of drug fever, eosinophil count was significantly elevated during the febrile episode. The possibility of the high fever (>39 °C) being solely due to severe pneumonia was ruled out as it was inconsistent with the patient’s overall infectious trajectory. Central fever originating from the patient’s unresolved traumatic brain injury was deemed unlikely given the prompt normalization of temperature upon PMB withdrawal (Khanum et al., 2024).

PMB is a hydrophilic molecule with a positive charge that possesses the ability to bind endotoxins. Its cationic N-terminal cyclic polypeptide binds electrostatically to the anionic lipopolysaccharide (LPS) in the outer membrane of Gram-negative bacteria and neutralizes free LPS, thereby reducing endotoxin activity and mitigating cytokine storm (Thomas and Surolia, 1999). The precise pathological mechanism behind PMB-induced drug fever remains unclear but may be related to intrinsic drug properties or formulation impurities. As a cationic cyclic polypeptide, PMB may trigger two immune pathways: 1) The hapten-type IV T-cell pathway: PMB may act as a hapten, conjugating with tissue proteins to form a complete antigen, leading to a type IV hypersensitivity reaction mediated by T-cells and the release of endogenous pyrogens such as interleukin-6 and TNF-α (Flanagan, 1971; Afra et al., 2025). 2) The Toll-like receptor 4 (TLR4)-mediated type I cytokine storm pathway: Residual LPS in the PMB formulation, LPS from massive bacterial lysis post-administration, or excipients may activate the TLR4 pathway, inducing a type I cytokine storm and triggering a systemic inflammatory response. PMB may also activate the MRGPRX2 receptor on mast cells, causing rapid release of histamine and leukotrienes, which aligns with and can amplify the cytokine storm via downstream TLR4 signaling, characteristic of a type I hypersensitivity reaction capable of causing rapid-onset high fever within 2 h (Chen and Wang, 2025; McCabe, 1973). The rapid onset of fever and its swift resolution upon discontinuation in this case, coupled with observed tachypnea following nebulized PMB, are more consistent with a classic type I hypersensitivity reaction. However, the fever duration exceeding 24 h does not preclude a potential mixed mechanism.

ADRs to PMB exhibit a “multi-system, dose-dependent, and time-related” pattern (Dubrovskaya et al., 2015). A review of case reports on PMB ADRs reveals that they primarily involve the skin and its appendages, particularly hyperpigmentation, followed by the nervous, urinary, respiratory, endocrine/metabolic, and musculoskeletal systems (Zhang et al., 2017; Chen and Wang, 2025). Dermatological reactions are the most frequently reported, while neurotoxicity and nephrotoxicity are the most severe, constituting the primary reasons for PMB’s restricted use. The fastest reported onset of an ADR was bronchospasm occurring 10 min after nebulization, leading to anaphylactic shock (Chin et al., 2024). Moreover, Wilson et al. documented a case of an asthmatic patient who developed acute respiratory failure requiring intubation within minutes after the initiation of PMB nebulization, directly implicating airway hyperreactivity as the underlying mechanism (Wilson, 1971). Both airway hyperreactivity and anaphylactic shock are associated with PMB-induced type I hypersensitivity. In our case, the patient exhibited tachypnea following nebulization, which aligns with the airway irritant effects of topical PMB described in these historical reports. Notably, the dual-route administration (intravenous plus nebulized PMB) could increase the total antigenic load. Unlike intravenous delivery alone, nebulized PMB directly exposes the airway mucosa, which is rich in mast cells, eosinophils, and other immunocompetent cells. This local exposure may not only trigger tachypnea but also exacerbate the systemic febrile response (Cao and Cao, 2023). This high-concentration local exposure may act as a “second hit,” not only inducing immediate bronchial irritation or type I hypersensitivity (manifesting as tachypnea) but also enhancing the overall sensitization burden. In susceptible individuals, this increased antigenic stimulation could lower the threshold for or amplify the severity of systemic hypersensitivity reactions, such as drug fever. A prior survey of 317 courses of colistimethate sodium (polymyxin E) reported drug fever in three of 72 adverse reaction episodes (4.17%) (Flanagan, 1971). For PMB, no specific case has been reported, but the actual potential for polymyxins to cause sensitization—particularly when administered via both systemic and topical routes—may be under-recognized. The seemingly low incidence of allergic reactions could be attributed to generally short treatment durations, limited prior exposure in most patients, or the fact that dual-route therapy has only recently gained wider clinical use (Zhang et al., 2025). Future pharmacokinetic and immunomonitoring studies should consider measuring PMB concentrations in both plasma and bronchoalveolar lavage fluid, as well as tracking eosinophil activation markers, to better define the relationship between local drug exposure and systemic hypersensitivity.

Given that PMB-induced ADRs can affect multiple organ systems, enhanced pharmaceutical vigilance is warranted in clinical practice, extending beyond severe reactions like anaphylactic shock to include challenging-to-diagnose conditions such as drug fever. The difficulty in distinguishing drug fever from the original infectious fever makes timely detection and management crucial. Drug fever should be confirmed and the causative agent discontinued as early as possible to alleviate patient discomfort and shorten unnecessary drug exposure. When drug fever due to an antimicrobial is suspected, an alternative agent with similar spectrum but a completely different molecular structure should be considered. For CRAB infections, newer agents like sulbactam-durlobactam or eravacycline may be options if available (Tamma et al., 2024). If no suitable alternative exists, prophylactic use of corticosteroids, non-steroidal anti-inflammatory drugs, or histamine receptor antagonists may be considered to prevent hypersensitivity reactions (Hama et al., 2022). Clinical pharmacists should conduct pharmaceutical care based on the patient’s medication history, integrating treatment progress, clinical symptoms, and laboratory findings. By assisting physicians in identifying drug fever, they can help ensure effective anti-infective therapy while avoiding waste of medical resources and reducing the patient’s economic burden. Only one case of PMB-induced drug fever has been reported so far, and the evidence remains limited. Further case reports would help enhance clinical awareness of this potential adverse effect.

## Data Availability

The original contributions presented in the study are included in the article/supplementary material, further inquiries can be directed to the corresponding authors.
